# Dissection of the Pre-Germinal Center B-Cell Maturation Pathway in Common Variable Immunodeficiency Based on Standardized Flow Cytometric EuroFlow Tools

**DOI:** 10.3389/fimmu.2020.603972

**Published:** 2021-02-17

**Authors:** Lucía del Pino-Molina, Eduardo López-Granados, Quentin Lecrevisse, Juan Torres Canizales, Martín Pérez-Andrés, Elena Blanco, Marjolein Wentink, Carolien Bonroy, Jana Nechvatalova, Tomas Milota, Anne-Kathrin Kienzler, Jan Philippé, Ana E. Sousa, Mirjam van der Burg, Tomas Kalina, Jacques J.M. van Dongen, Alberto Orfao

**Affiliations:** ^1^ Clinical Immunology Department, La Paz University Hospital and Lymphocyte Pathophysiology in Immunodeficiencies Group, La Paz Institute for Health Research (IdiPAZ) and Center for Biomedical Network Research on Rare Diseases (CIBERER U767), Madrid, Spain; ^2^ Clinical and Translation Research Program, Cancer Research Centre (IBMCC, USAL-CSIC), Department of Medicine, Cytometry Service (NUCLEUS), University of Salamanca (USAL), Institute of Biomedical Research of Salamanca (IBSAL), Salamanca, Spain; ^3^ Biomedical Research Networking Centre Consortium of Oncology (CIBERONC) Instituto de salud Carlos III, Madrid, Spain; ^4^ Department of Immunology, Erasmus University Medical Center (Erasmus MC), Rotterdam, Netherlands; ^5^ Department of Laboratory Medicine, University Hospital Ghent, Ghent, Belgium; ^6^ Department of Allergology and Clinical Immunology, Faculty of Medicine, Masaryk University and St Anne’s University Hospital in Brno, Brno, Czechia; ^7^ Department of Immunology, Second Faculty of Medicine, Charles University and Motol University Hospital, Prague, Czechia; ^8^ Nuffield Department of Medicine, Experimental Medicine Division, University of Oxford, Oxford, United Kingdom; ^9^ Instituto de Medicina Molecular, Faculdade de Medicina, Universidade de Lisboa, Lisboa, Portugal; ^10^ Department of Pediatrics, Laboratory for Immunology, Leiden University Medical Center, Leiden, Netherlands; ^11^ CLIP - Childhood Leukemia Investigation Prague, Department of Pediatric Hematology and Oncology, 2nd Faculty of Medicine, Charles University and University Hospital Motol, Prague, Czechia; ^12^ Department of Immunohematology and Blood Transfusion, Leiden University Medical Center (LUMC), Leiden, Netherlands

**Keywords:** CVID, Pre-GC B-cell tube, pre-GC maturation pathway, expression markers, EuroFlow standardization

## Abstract

**Introduction:**

Common Variable Immunodeficiency (CVID) is characterized by defective antibody production and hypogammaglobulinemia. Flow cytometry immunophenotyping of blood lymphocytes has become of great relevance for the diagnosis and classification of CVID, due to an impaired differentiation of mature post-germinal-center (GC) class-switched memory B-cells (MBC) and severely decreased plasmablast/plasma cell (Pb) counts. Here, we investigated in detail the pre-GC B-cell maturation compartment in blood of CVID patients.

**Methods:**

In this collaborative multicentric study the EuroFlow PID 8-color *Pre-GC B-cell tube*, standardized sample preparation procedures (SOPs) and innovative data analysis tools, were used to characterize the maturation profile of pre-GC B-cells in 100 CVID patients, vs 62 age-matched healthy donors (HD).

**Results:**

The *Pre-GC B-cell tube* allowed identification within pre-GC B-cells of three subsets of maturation associated immature B-cells and three subpopulations of mature naïve B-lymphocytes. CVID patients showed overall reduced median absolute counts (vs HD) of the two more advanced stages of maturation of both CD5^+^ CD38^+/++^ CD21^het^ CD24^++^ (2.7 vs 5.6 cells/µl, p=0.0004) and CD5^+^ CD38^het^ CD21^+^ CD24^+^ (6.5 vs 17 cells/µl, p<0.0001) immature B cells (below normal HD levels in 22% and 37% of CVID patients). This was associated with an expansion of CD21^-^CD24^-^ (6.1 vs 0.74 cells/µl, p<0.0001) and CD21^-^CD24^++^ (1.8 vs 0.4 cells/µl, p<0.0001) naïve B-cell counts above normal values in 73% and 94% cases, respectively. Additionally, reduced IgMD^+^ (21 vs 32 cells/µl, p=0.03) and IgMD^-^ (4 vs 35 cells/µl, p<0.0001) MBC counts were found to be below normal values in 25% and 77% of CVID patients, respectively, always together with severely reduced/undetectable circulating blood pb. Comparison of the maturation pathway profile of pre-GC B cells in blood of CVID patients vs HD using EuroFlow software tools showed systematically altered patterns in CVID. These consisted of: i) a normally-appearing maturation pathway with altered levels of expression of >1 (CD38, CD5, CD19, CD21, CD24, and/or smIgM) phenotypic marker (57/88 patients; 65%) for a total of 3 distinct CVID patient profiles (group 1: 42/88 patients, 48%; group 2: 8/88, 9%; and group 3: 7/88, 8%) and ii) CVID patients with a clearly altered pre-GC B cell maturation pathway in blood (group 4: 31/88 cases, 35%).

**Conclusion:**

Our results show that maturation of pre-GC B-cells in blood of CVID is systematically altered with up to four distinctly altered maturation profiles. Further studies, are necessary to better understand the impact of such alterations on the post-GC defects and the clinical heterogeneity of CVID.

## Introduction

Common Variable Immunodeficiency (CVID) is the most prevalent symptomatic primary immunodeficiency (PID). It is characterized by defective antibody production that leads to hypogammaglobulinemia (1–3) with an increased susceptibility to infections, associated in some CVID patients with enteropathy, autoimmunity, lymphoproliferation, and/or risk of lymphoid malignancy due to more profound immunological dysregulation (4, 5). Despite distinct monogenic defects are present in a minor fraction (<20%) of cases, and other complex oligo or polygenic genetic predisposition (6), and epigenetic alterations (e.g., impaired demethylation in genes relevant for the B cell functions) have been associated with the development of CVID (7), a well-defined pathogenic mechanism still remains to be identified in most CVID patients. Thus, assessment of the distribution of lymphocytes, particularly post-germinal center (GC) B-cells and plasmablasts/plasma cells in blood of suspicious patients by flow cytometry has become of great relevance for the diagnosis and classification of CVID (8, 9).

Impaired post-GC B cell maturation in the periphery (i.e., in blood and secondary lymphoid tissues) is a hallmark of CVID. However, CVID is a rather heterogeneous disease from the clinical, genetic and immunologic point of view. Thus, several classification algorithms have been proposed for CVID, which are based on the specific alterations encountered for the major B cell populations in blood (8), their proliferation history and somatic hypermutation levels (9), in combination or not with the clinical manifestations of the disease and/or more sophisticated computational (i.e., hierarchical clustering) approaches (10). Overall, impaired differentiation of mature post-GC B-cells, consisting of severely reduced circulating class-switched memory B-cells (MBC) and strongly decreased (i.e., undetectable) plasmablast/plasma cell production, are the most consistent defects in CVID. Because of this, demonstration of reduced class-switched MBC is now used among the diagnostic criteria proposed by the European Society for Immunodeficiencies (ESID) for CVID (11). In turn, depending on the specific defects encountered in the post-GC MBC and Pb compartments in blood, and the severity of such defects, distinct CVID patient subgroups, associated with distinct clinical profiles, have also been identified (12).

Apart from the alterations in post-GC B-cells and plasmablasts/plasma cells, an increasing number of evidences indicate that the production and maturation of B-cells in bone marrow (BM) is also altered in at least a fraction (e.g., around one third) of all CVID patients due to either a maturation blockade (13) and/or an altered bone marrow environment which is non-permissive for B-cell maturation (14). Thus, a significant proportion of CVID patients display reduced absolute B-cell counts in blood (5) and an early B-cell maturation arrest in BM (15). In addition, expansion of transitional/immature B cells, and CD21^low^ B-cells has also been reported in a subset of CVID patients (8, 16). Altogether, these findings further support an impaired maturation of pre-GC B-cells in CVID.

Herein, we investigated in detail the pre-GC B-cell maturation compartment in blood of 100 CVID patients, taking advantage of the novel and standardized flow cytometric approaches developed by EuroFlow for this purpose (17–19), e.g., the recently proposed EuroFlow PID 8-color antibody panels (20) that can be easily implemented in most diagnostic laboratories worldwide, together with the EuroFlow standard operating procedures (SOPs) for sample preparation, data acquisition and analysis, including innovative data analysis tools recently developed by EuroFlow to assess normal vs altered pre-GC B-cell maturation profiles in blood (17, 20–23).

## Material and Methods

### Patients, Controls, and Samples

Overall, 100 adult CVID patients -50 men and 50 women; median age: 41 years (y); range: 16–82y- and 62 healthy donors (HD) not related to the patients (33 men and 29 women; median age: 34y; range: 19–67y) were studied in parallel, at eight different EuroFlow-PID centers. CVID was diagnosed locally at each center, according to the ESID criteria (3, 24). Relevant clinical data on CVID patients was obtained from the patients’ health electronic records or from national patient registries and collected at each of the 8 participating centers, including data on: patient age, gender, immunoglobulin (Ig) levels and response to vaccination at diagnosis, together with data on prior infections and type of infections (e.g., upper and lower bacterial respiratory infections, viral and fungal infections), autoimmunity (e.g., cytopenias, organ-based and systemic auto-immunity), lymphoproliferation, lymphoid interstitial pneumonitis (LIP), granulomas, splenomegaly, hepatomegaly, bronchiectasias, enteropathy, and malignancy, as well as prior therapy, including Ig replacement therapy.

Blood samples were obtained, processed and measured by flow cytometry at each of the 8 participating centers after informed consent had been given by each individual participant, according to the principles of the Declaration of Helsinki. The study was approved by the local Ethics Committees of the participating centers: Hospital Universitario La Paz, Madrid, Spain (PI-2833 and 2009/3348/I); Charles University, Prague, Czech Republic (15-28541A); Erasmus MC, Rotterdam, The Netherlands (MEC-2013-026); St Anne´s University, Brno, Czech Republic (METC 1G2015); BRC-Translational Immunology Lab, University of Oxford, Oxford, United Kingdom; University of Salamanca, Salamanca, Spain (USAL/CSIC 20-02-2013); University Hospital of Ghent, Belgium (B670201523515); and Faculdade de Medicina da Universidade de Lisboa and Centro Hospitalar Universitário Lisboa Norte, Lisbon, Portugal (937/13).

### Flow Cytometric Identification of B-Cells, Plasmablasts/Plasma Cells and Their Subsets in Blood

Blood samples from both CVID patients and HD were processed and stained at each center with the EuroFlow 8-color *PIDOT* and *Pre-GC B-cell* tubes, following the EuroFlow SOPs for staining of cell surface membrane (sm) markers only, as previously described (20–22). Details about the specific antibody clones and fluorochrome-conjugated reagents used are provided in **Supplementary Table 1**. Instrument set-up and calibration were performed prior to data acquisition on ≥1 x 10^6^ cells (range: 1 x 10^6^-5 x 10^6^ cells) in FACSCanto II flow cytometers −Becton/Dickinson Biosciences (BD), San José, CA-, following the EuroFlow SOPs available at www.EuroFlow.org (21). Data analysis was performed centrally on pseudoanonymazed flow cytometry standard (FCS) data files deposited in the EuroFlow data repository, using the *Infinicyt* software (Cytognos SL, Salamanca, Spain).

For data analysis, a standardized gating strategy was used for identification of all pre-GC (defined as CD19^+^ CD27^-^ sIgM^+^ B-lymphocytes) and post-GC B-cell subsets (defined as CD19^+^ CD27^+^ or CD19^+^ CD27^-^ smIgM^-^ B-cells) present in blood, based on the EuroFlow-PID *Pre-GC B cell tube* as illustrated in **Supplementary Figure 1**. Briefly, CD19^+^ B-cells and plasmablasts/plasma cells were both identified by their low-to-intermediate forward (FSC) and sideward (SSC) light scatter properties after excluding debris and cell doublets. Subsequently, both cell subsets were sub-classified into 11 different subsets based on their staining profile for CD19, CD38, CD24, CD21, CD27, CD5, smIgM, and smIgD: a) CD27^-^ CD38^hi^ CD24^hi^ CD5^+^ smIgM^++^D^+^ immature/transitional B cells; b) CD27^-^ CD38^-^ CD24^het^ CD5^het^ smIgM^+^IgD^++^ mature naive B lymphocytes; c) CD27^+^ CD38^lo^ CD5^-^ CD24^het^ smIgM^++^D^+^ (MD^+^) unswitched MBCs; d) CD27^+/-^ CD38^lo^ CD5^-^ CD24^het^ smIgM^-^D^-^ (MD^-^) switched MBCs; and, e) CD27^++^ CD38^hi^ CD5^-^ CD21^-^ CD24^-^ plasmablasts/PCs. Immature/transitional B cells were further sub-classified according to the pattern of expression of CD38, CD5, CD21, and CD24 into three subsets of increasingly more mature B-lymphocytes: a1) CD5^-^ CD38^++^ CD21^het^ CD24^++^; a2) CD5^+^ CD38^+/++^ CD21^het^ CD24^++^, and a3) CD5^+^ CD38^het^ CD21^+^ CD24^+^ immature/transitional B lymphocytes. In turn, mature naive B-lymphocytes and unswitched MBCs were also further sub-classified into three subsets each, based on the expression profile for CD21 and CD24, into CD21^+^ CD24^+^, CD21^-^ CD24^++^, and CD21^-^ CD24^-^ mature naïve B cells and unswitched MBC, respectively.

For each B cell population, absolute counts were calculated using a dual platform assay based on the white blood cell (WBC) count, as assessed in a conventional hematological cell counter, and the percentage of total B cells obtained with the *PIDOT* tube for the same sample, as previously reported (22). Normal reference ranges were defined by the 5^th^ and 95^th^ percentile values observed in blood of 62 (age- and sex-matched) HD analyzed in parallel to the CVID patients (**Supplementary Table 2**).

### Pre-GC B-Cell Maturation Pathway in Blood

**Figure 1:**
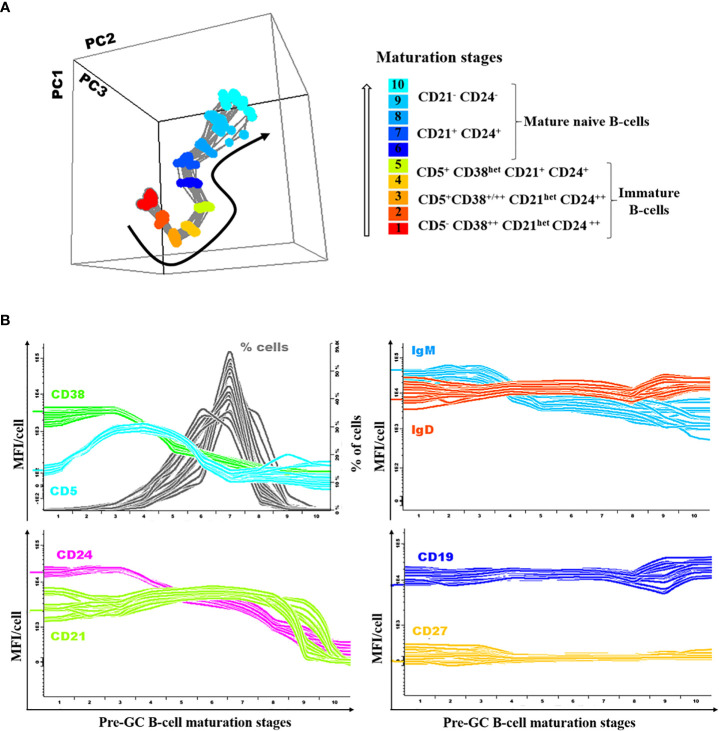
Illustrating example of the normal (reference) pre-germinal-center (GC) maturation pathway. Overall phenotypes **(A)** and expression levels for individual markers per stage of maturation **(B)** are shown for each individual donor (n=18) included in the normal reference pre-GC B-cell maturation pathway used to define reference normal values, depicted as color-coded lines per marker/parameter.

A database reflecting the normal B-cell maturation pathway of pre-GC B-lymphocytes in blood was built by merging data files from 18 representative HD, using the *Infinicyt* software (Cytognos SL) and previously described procedures (25). For this purpose, pre-gated data files which specifically contained gated data exclusively on the three different subsets of immature blood B cells (CD5^-^ CD38^++^ CD21^het^ CD24 ^++^, CD5^+^ CD38^+/++^ CD21^het^ CD24^++^ and CD5^+^ CD38^het^ CD21^+^ CD24^+^ immature B cells), together with both the CD21^+^ CD24^+^ and CD21^-^ CD24^-^ mature naive B cell subsets, from blood of 18 HD stained with the *Pre-GC B-cell* tube were merged into a single data file. Mature naive CD21^-^ CD24^++^ B cells were not included in the pre-GC B-cell database since this subset is barely detectable in normal blood from HD (26). Then, the merged data file was used to define the maturation pathway of pre-GC B-cells using the maturation tool developed by EuroFlow and implemented in Infinicyt (v2.0-4b for EuroFlow members only). This tool allows for automatic i) definition of vectors that reflect maturation pathways based on curve analysis algorithms, ii) classification of events into different maturation stages arbitrarily set at equal distances, iii) calculation of descriptive statistics for all events classified within each maturation stage, followed by direct visualization in a (balanced) 3-dimension (3D) APS (automated population separator) diagram, constructed using the first three principal components (PC1 to PC3) derived from PC analysis (PCA) performed with the *Infinicyt* software (**Figure 1A**). Thus, based on the maturation tool of the *Infinicyt* software, 10 distinct pre-GC B-cell maturation stages were (arbitrarily) defined and the normal mean fluorescence intensity (MFI) range (2SD) per maturation stage was calculated for each individual cell surface marker. Afterward, deconvolution of the maturation vector defined by the median MFI values per maturation stage (n=10) data was plotted for the whole set of phenotypic markers included in the EuroFlow *Pre-GC B-cell tube* (**Figure 1A**). Subsequently, median (range) and mean (+2 SD) fluorescence intensity (MFI) values per marker in HD blood pre-GC B-cells together with the corresponding B cell percentage, were plotted along the different maturation stages and used as normal reference range (**Figure 1B**). Subsequently, gated pre-GC B-cells from every individual CVID case were plotted against the normal reference maturation database and differences in MFI values vs. the normal blood range were recorded per marker for each of the 10 pre-established pre-GC B-cell maturation stages. MFI values below 2SD or above 2SD of the normal reference range (per maturation stage) for at least two consecutive stages of maturation of pre-GC B-cells, were considered to be altered. In parallel, a graphical display of the values per marker along the pre-GC B-cell maturation pathway, in which each patient is represented against the database, was obtained.

### Statistical Analyses

Statistical analyses were performed with GraphPad Prism Software version 6.0 (Graph- Pad software, San Diego, CA). To define normal ranges for each B cell subset identified in blood, 5^th^ and 95^th^ percentile values from the 62 adult HD were used. Group comparisons were performed using the Mann-Whitney U and Kruskal-Wallis tests (for continuous variables) or the Fisher exact and X^2^ tests (for categorical variables). Clustering analysis based on K-means was performed using the JMP software (free trial version 14; SAS Institute Inc., Cary, NC) based on the immunophenotypic profiles and relative distributions of pre-GC B-cells along the pre-GC maturation pathway, per maturation stage. Cluster analysis was performed by simultaneously comparing the MFI values for each surface marker and the percentage of events per stage of maturation per CVID patient against the maturation reference database. P-values<0.05 were considered to be associated with statistical significance and coded hereafter as follows: *p-value<0.05; ** p-value<0.01; *** p-value<0.001; and, **** p-value<0.0001.

## Results

### Distribution of Pre-GC B-Cell Subsets in Blood of CVID Patients

**Figure 2:**
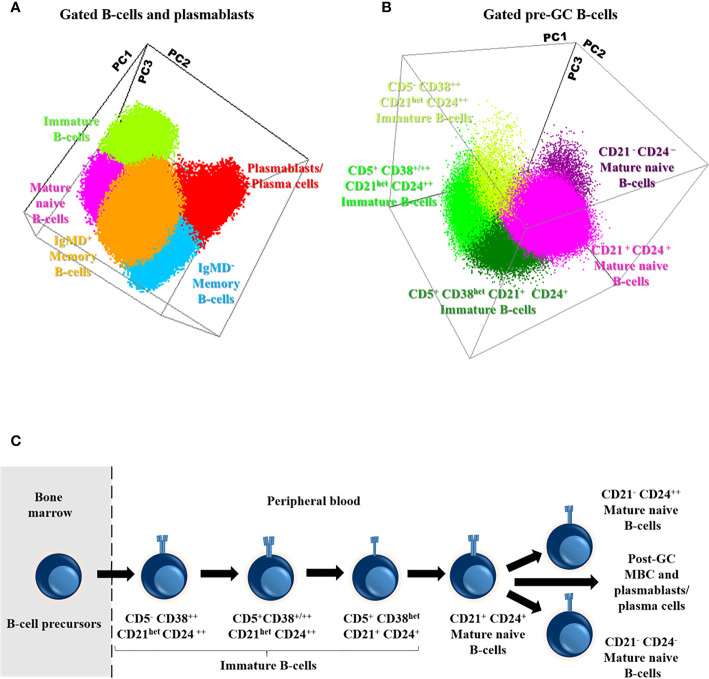
Distribution of the major subsets of immature, naive and memory B cells (MBC) and plasmablasts/plasma cells **(A)**, including the different subsets of pre-germinal-center (GC) B-cells **(B)**, in blood of a representative adult healthy donor (HD) and their maturation-associated relationship **(C)**. Three-dimensional principal component (PC) analysis (PCA) plots in **(A, B)** were built based on PC1 (A: mean fluorescence intensity of CD27, 29.25%; IgD, 22.56%; IgM, 20.45%; CD21, 11.16%; CD38, 9.63% and CD5, 6.95%; B: mean fluorescence intensity of CD21, 30.15%; CD24, 27.64%; CD38, 23.07% and CD5, 19.14%), PC2 (A: mean fluorescence intensity of IgM, 43.89%, CD27, 34.62%, CD38, 10.18%, IgD, 9.96%; CD21, 1.16% and CD5, 0.18%; B: mean fluorescence intensity of CD21, 43.66%, CD38, 23.62%, CD24, 17.09% and CD5, 15.63%) and PC3 (**A**: mean fluorescence intensity of CD38, 42.02%, CD21, 19.56% CD27, 12.19%, CD5, 12.03%, IgD, 11% and IgM, 3.20%; **B**: mean fluorescence intensity of CD5, 56.16%, CD24, 27.80%, CD38, 14.85% and CD21, 1.19%) vectors using the (balanced) automated population separator (APS1-2) 3-D view of Infinicyt. In both **(A, B)**, the distinct color-coded cell populations displayed were gated as described in **Supplementary Figure 1**.

Based on the data provided by the *PID-orientation tube (PIDOT) *(22), and the *Pre-GC B-cell* tube, detailed characterization of B cells in peripheral blood was achieved for a total of 11 distinct B cell subsets: i) immature/transitional B-cells (including three maturation-associated populations of CD5^-^ CD38^++^ CD21^het^ CD24 ^++^, CD5^+^ CD38^+/++^ CD21^het^ CD24^++^, and CD5^+^ CD38^het^ CD21^+^ CD24^+^ immature B-cells); ii) mature naïve B-cells (and their three CD21^+^ CD24^+^, CD21^-^ CD24^++^, and CD21^-^ CD24^-^ subsets); iii) unswitched IgMD^+^, IgM^+^-only, and IgD^+^ -only MBC (and their CD21^+^ CD24^+^, CD21^-^ CD24^-^, and CD21^-^ CD24^++^ subsets); iv) switched IgMD^-^ MBC; and v) plasmablasts/plasma cells (**Figures 2A–C**).

**Figure 3:**
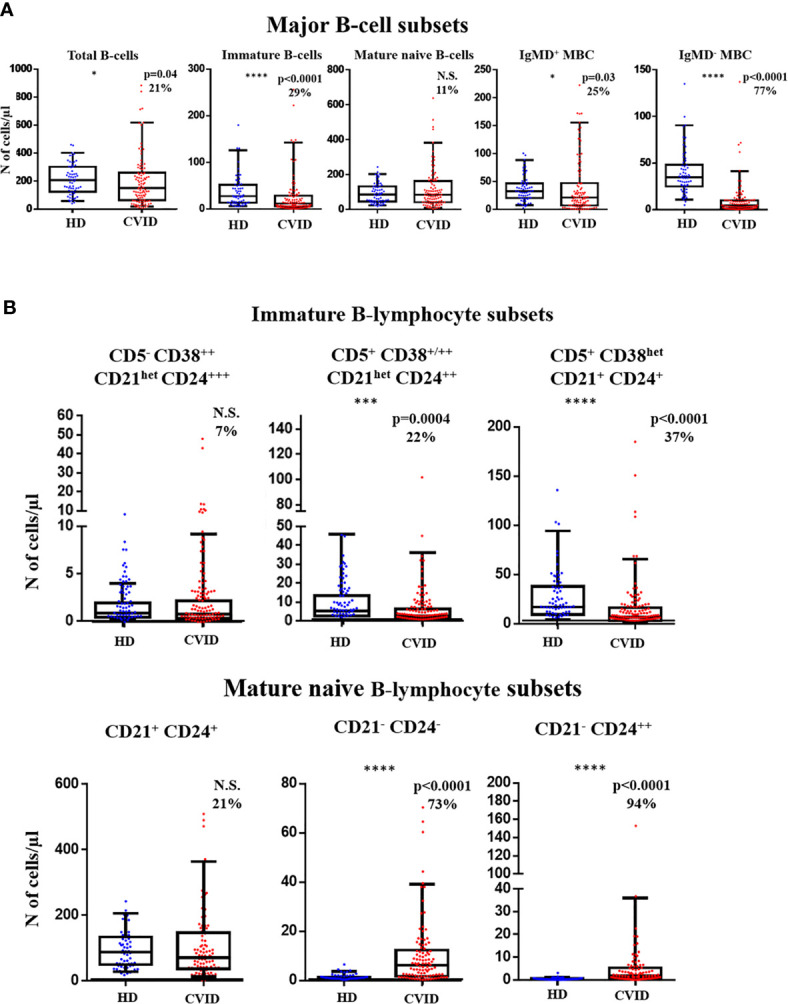
Distribution of distinct subsets of pre-germinal-center (GC) and post-GC B-cells in blood of Common Variable Immunodeficiency (CVID) patients (n=100) vs age-matched HD (n=62). **(A)** absolute counts of major pre-GC and post-GC B-cell subsets are shown using box and whiskers plots separately for healthy donor (HD) (n=62) and CVID patients (n=100) where horizontal lines and vertical lines represent the median and both 5^th^ and 95^th^ percentile values, respectively. Percentage of CVID patients with decreased or increased counts below or above normal values (<5^th^ and **>**95^th^ percentile) detected in age-matched HD are shown as percent values. **(B)** absolute pre-GC CD5^-^ CD38^++^ CD21^het^ CD24^+++^, CD5^+^ CD38^+/++^ CD21^het^ CD24^++^, and CD5^+^ CD38^het^ CD21^+^ CD24^+^ immature B-cell and CD21^+^CD24^+^, CD21^-^CD24^-^, and CD21^-^ CD24^++^ naïve B-cell subset counts in CVID vs HD. N.S. not statistically significant differences detected, *p-value<0.05; ***p-value<0.001; ****p-value<0.0001 (Mann Whitney U test).

Overall, the total B-cell count in blood of CVID was significantly reduced vs. age-matched HD (median: 149 vs 206 cells/µl; p=0.04). Such decrease was mostly due to a significant reduction of immature B-cells (11 vs 27 cells/µl, p<0.0001), IgMD^+^ MBC (21 vs 32 cells/µl, p=0.03) and particularly, IgMD^-^ MBC counts (4 vs 35 cells/µl, p<0.0001), in the absence of virtually no plasmablasts/plasma cells (**Figure 3A**). Despite the overall reduced median B-cell counts observed in CVID, a significant overlap with HD was still observed with variable frequencies and patterns of alteration among CVID patients. Thus, decreased counts below normal values (<5^th^ percentile of age-matched HD) of IgMD^-^ MBC were detected in 77% of the CVID patients investigated, together with undetectable plasmablasts/plasma cells in 100% of cases. In contrast, reduced immature/transitional B cells and IgMD^+^ MBC counts were only found in 29% and 25% of cases, respectively (**Figure 3A**). In turn, only a small percentage of all CVID patients showed reduced mature naïve B-cell counts (11%). Altogether, the decreased numbers of the distinct B-cell subsets led to overall low total B-cell counts in blood of 21% of all CVID patients (**Figure 3A**).

More detailed analysis of the pre-GC B-cell compartment also showed distinct patterns of alteration for different subsets of immature B-cells and mature naive B-cells. Thus, reduced counts (vs age-matched HD) of the more advanced stages of maturation of CD5^+^ CD38^+/++^ CD21^het^ CD24^++^ (2.7 vs 5.6 cells/µl, p=0.0004) and CD5^+^ CD38^het^ CD21^+^ CD24^+^ (6.5 vs 17 cells/µl, p<0.0001) immature/transitional B cells was detected in 22% and 37% of CVID patients. In contrast, the less differentiated CD5^-^ CD38^++^ CD21^het^ CD24 ^++^ immature/transitional B lymphocytes (0.79 vs 0.89, p>0.05) were decreased in blood in only 7% of CVID patients (**Figure 3B**). Regarding mature naive B-cells, an increase in CD21^-^CD24^-^ (6.1 vs 0.74 cells/µl, p<0.0001) and CD21^-^ CD24^++^ (1.8 vs 0.4 cells/µl, p<0.0001) naive B cell counts was observed in CVID vs. HD, with a clear bimodal distribution (**Figure 3B**) due to the presence of a major subgroup of patients (73% and 94%, respectively) presenting a significant expansion of these two naive B-cell subsets, in association or not with low CD21^+^ CD24^+^ naïve B-cell counts, which were found to be reduced in only 21% of CVID patients (**Figure 3B**).

Regarding post-GC MBC, CVID patients displayed a significant reduction (vs. HD) of CD21^+^ CD24^+^ IgMD^+^ MBC (16 vs 31 cells/µl, p=0.0006), associated with normal or slightly increased (p>0.05) CD21^-^CD24^-^ and CD21^-^ CD24^++^ IgMD^+^ MBC numbers (**Supplementary Figure 2**). Plasmablasts/plasma cells, were either not detected or severely reduced in 100% of CVID patients. Altogether, these results suggest the existence of different maturation blockades and profiles in CVID, which frequently also involve pre-GC B-cells, in addition to post-GC MBC and plasmablasts/plasma cells.

### Pre-GC B-Cell Maturation Profile in Normal Blood

Based on the innovative maturation tools developed by EuroFlow (18, 25), a normal pre-GC B-cell reference maturation pathway was built as described above in the material an methods section and illustrated in **Figure 1A**.

Detailed analysis of the normal pre-GC B-cell maturation profile showed downregulation of CD38 from stage 3 on, associated with strong CD5 and CD24 expression at early stages (stages 2–4 and stages 1–3, respectively), CD5 becoming negative from stage 7 onwards while CD24 progressively decreased from stage 4 onward; in turn, CD21 was strongly expressed from stage 1 until the last stages of maturation (stages 9 and 10), when it was downregulated. Among the other markers investigated, smIgM showed slightly higher expression levels at early (stages 1–3) vs later stages with stable levels from stage 4 to 10, while IgD and CD19 showed similarly stable expression levels along all stages of maturation of pre-GC B-cells. By definition CD27 was not expressed in pre-GC B-cells (**Figure 1B**).

### Altered Maturation Profiles of Blood Pre-GC B-Cells in CVID

**Figure 4:**
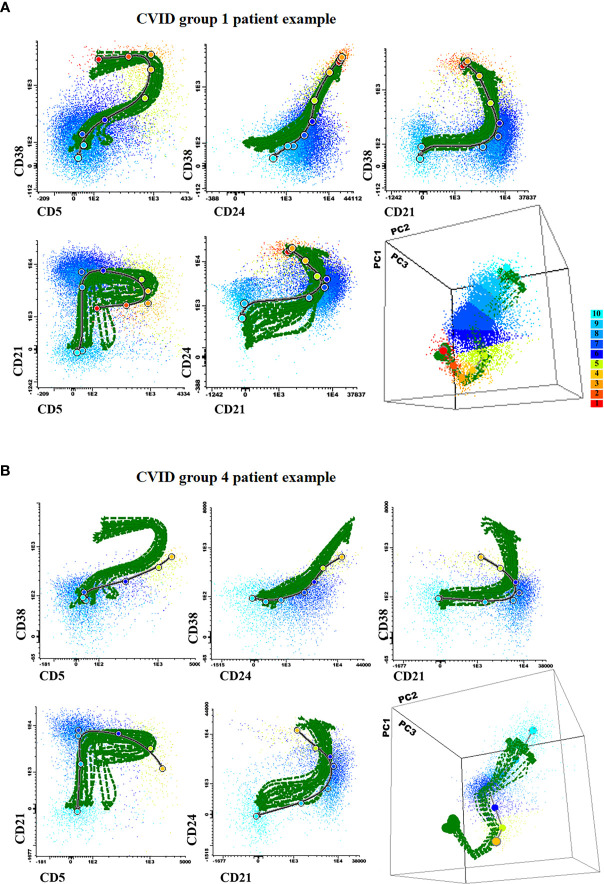
Illustrating 2-dimensional and 3-dimensional dot plot graphical examples of the maturation pathway of pre-GC B-cells in representative Common Variable Immunodeficiency (CVID) patients showing normal-appearing (**A**; CVID groups 1–3) vs severely disturbed maturation pathways (**B**; CVID group 4). In all plots, the reference pre-germinal-center (GC) B-cell maturation pathway defined for 18 (individual) HD green lines is shown together with a gray/black line corresponding to the maturation pathway of the two individual CVID patients shown in **(A, B)**, respectively. Colored dots correspond to the median values obtained for the 10 different maturation stages (color code defined in the right) where stage 1 is colored as blue and stage 10 is depicted in red.

**Figure 5:**
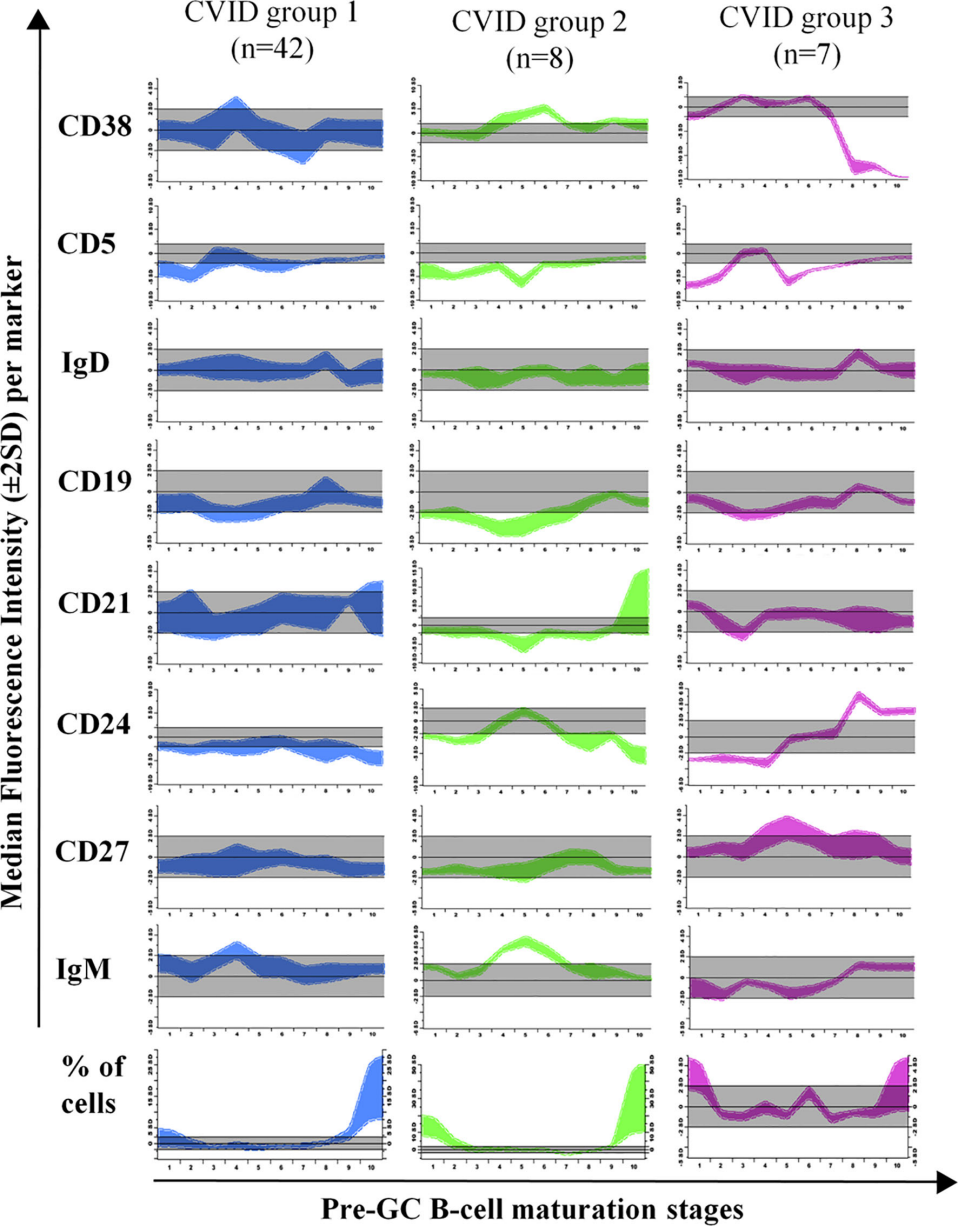
Maturation diagrams per marker of all Common Variable Immunodeficiency **(**CVID**)** patients included in each of the three CVID groups of patients displaying a normal-appearing pre-GC maturation profile in blood (groups 1–3). In each diagram the (normalized) reference maturation pathway is shown as a grey area for the ±2 SD of normal MFI values per marker. The pre-GC B-cell maturation profile found in CVID patients from each group is displayed as ±2 SD bars color-coded by CVID patient group (CVID group 1 is shown in blue, group 2 in green and group 3 in pink). Values below or above the normal 2SD limit for ≥ 2 consecutive maturation stages were considered to reflect an altered marker expression profile.

Direct comparison of the phenotype of maturation-associated blood pre-GC B-cell subsets from CVID patients vs. the normal maturation was performed in 88 patients by plotting phenotypic data from each CVID against the normal reference maturation database (**Figures 4** and **5**). For better visualization of the phenotypic deviations from normal, a normalized scale (**Supplementary Figure 3**) was used. Overall, distinct patterns of alteration (cell counts and/or MFI values per marker in ≥2/10 consecutive stages of maturation of pre-GC B-cells falling ≥2 SD apart from the normal distribution) of the pre-GC B-cell maturation were detected in every case (88/88; 100%). Thus, four distinct patterns of alterations were identified: a) CVID with normally-appearing maturation pathways (groups 1 to 3) but distinct patterns of alteration on the levels of expression of individual phenotypic markers (57/88, 65%) and b) CVID patients with a clearly altered pre-GC B-cell maturation pathway (31/88 cases, 35%; CVID group 4) (**Figures 4A** and **B**). In turn, unsupervised clustering analysis further revealed the presence of three different profiles among the former CVID patient groups 1 to 3, depending on the pattern of deviation in the number of cells per maturation stage and the levels of expression of individual markers, from the normal reference maturation pathway (**Figure 5** and **Supplementary Figure 3**).

In group 1 a large fraction of CVID patients (42/88, 48%) was included, who showed significantly reduced total B-cell (128 vs 206 cells/µl, p=0.008) and both CD5^+^ CD38^+/++^ CD21^het^ CD24^++^ (2.6 vs 5.6 cells/µl, p=0.0013) and CD5^+^ CD38^het^ CD21^+^ CD24^+^ (7.3 vs 17 cells/µl, p<0.0001) immature/transitional B-cell numbers, in the absence of overall relevant phenotypic deviations from the normal maturation profile of blood pre-GC B-cells (**Supplementary Figure 3**). Group 2 consisted of 8/88 (9%) CVID patients that showed overexpression of smIgM at intermediate stages of maturation of pre-GC B-cells and of CD38 at early and late stages of maturation of pre-GC B-cells; compared to HD, CVID cases classified in group 2 also showed significantly higher counts of the most immature CD5^-^ CD38^++^ CD21^het^ CD24 ^++^ (7.6 vs 0.89 cells/µl, p=0.0009) pre-GC B-cell subset, associated with reduced numbers of more differentiated CD5^+^ CD38^het^ CD21^+^ CD24^+^ immature B lymphocytes (5.1 vs 17 cells/µl, p=0.0183) and IgMD^+^ (particularly CD21^+^ CD24^+^) MBC (4 vs 31 cells/µl, p<0.0001); in addition, group 2 patients also showed decreased levels of CD21 at stages 7–8, reflecting the parallel increase in CD21^-^ CD24^-^ naïve B cell counts (8.8 vs 0,74 cells/µl, p=0.02) (**Table 1**). Finally, group 3 included 7/88 (8%) CVID patients characterized by showing under-expression of CD38 together with higher levels of CD24 at the last stages of maturation of pre-GC B-cells, in line with the underlying increased counts of CD21^+^CD24^+^ mature naïve B cells observed among these cases vs. the other CVID patient groups 1 to 3: 137 vs 76, 42, and 59 cells/µl in CVID groups 1, group 2, and group 4, respectively (**Table 1**).

**Table 1:** Distribution of different subsets of blood B-cells in HD and CVID patients classified according to the presence of a normal-appearing (CVID groups 1-3) vs severely disturbed maturation pathway (CVID group 4).

B-cell subset	CVID patients group					p-value (vs HD)	p-value (among CVID groups)
	HD	Group 1(n=42)	Group 2(n=8)	Group 3(n=7)	Group 4(n=31)		
Total B-cells	206 (57.2-402)	128 (19-444)	132 (0.75-122)	222 (59-349)	180 (12-786)	0.0082^a^	NS
Immature B-cells	27 (5.7-126)	13 (1.6-54)	27 (5.2-222)	19 (6.1-41)	7.1 (0.43-191)	<0.0001^a^ <0.0001^d^	NS
CD5^-^CD38^++^ CD21^het^CD24 ^++^	0.89 (0.13-4)	0.98 (0.17-8.7)	7.6 (0.91-52)	1.6 (0.75-8.4)	0.36 (0-5.7)	0.0009^b^ 0.004^d^	0.0016^e^ 0.015^f^ <0.0001^h^ 0.0032^i^
CD5^+^ CD38^+/++^ CD21^het^ CD24^++^	5.6 (1.1-35)	2.6 (0.28-17)	12 (1.2-105)	3 (1.2-8.1)	1.4 (0-54)	0.001^a^ <0.0001^d^	0.04^e^ 0.033^f^ 0.0117^h^
CD5^+^ CD38^het^ CD21^+^ CD24^+^	17 (4-94)	7.3 (0.55-33)	5.1 (1.5-66)	7.7 (1.6-29)	5.3 (0.21-161)	<0.0001^a^ 0.018^b^ <0.0001^d^	NS
Mature naive B-cells	85 (24-203)	79 (8.5-313)	77 (10-221)	138 (25-285)	85 (9.6-532)	NS	NS
CD21^+^ CD24^+^	84 (23-201)	76 (7.6-215)	42 (9.7-197)	137 (22-264)	59 (4.6-419)	NS	NS
CD21^-^CD24^-^	0.74 (0.02-3.7)	4 (0.33-30)	8.8 (0-60)	10 (0.76-39)	9.9 (0.13-66)	<0.0001^a^ 0.02^b^ <0.0001^c^ <0.0001^d^	0.0018^f^
CD21^-^CD24^++^	0.4 (0-0.14)	1.5 (0.15-12)	4.2 (0-15)	0.62 (0.4-4.3)	3.5 (0.17-95)	<0.0001^a^ 0.003^b^ 0.008^c^ <0.0001^d^	0.023^f^
Memory B cells MBC	72 (19-159)	32 (1.2-165)	9.2 (0.9-26)	31 (19-155)	37 (1.4-372)	<0.0001^a^ <0.0001^b^ 0.027^c^	0.008^e^ 0.0006^g^ 0.013^h^
IgMD^+^ MBC	32 (7.6-88)	25 (0.91-127)	6.6 (0.35-14)	22 (8.1-144)	32 (0.81-364)	<0.0001^b^	0.007^e^ 0.0012^g^ 0.0051^h^
CD21^+^CD24^+^	31 (5.6-84)	18 (0.75-122)	4 (0.33-7.6)	21 (7-46)	18 (0.47-141)	0.014^a^ <0.0001^b^	0.0036^e^ 0.0006^g^ 0.0074^h^
CD21^-^CD24^-^	0.66 (0.13-3.8)	0.71 (0-5.7)	0.73 (0-3.1)	1.4 (0-95)	1.15 (0.02-50)	0.016^d^	NS
CD21^-^CD24^++^	1.18 (0.05-5)	2 (0.01-20)	0.8 (0-3.5)	1 (0-3)	4.1 (0-259)	0.003^d^	0.04^h^ 0.04^i^
IgMD^-^ MBC	35 (11-90)	4.1 (0.01-64)	1.2 (0-18)	11 (8.3-20)	4 (0.11-50)	<0.0001^a^ <0.0001^b^ <0.0001^c^ <0.0001^d^	0.0093^g^ 0.0062^i^

Results expressed as median absolute counts (5^th^-95^th^ percentiles) per B-cell subset. The Kruskal-Wallis and Mann-Whitney U tests were used with significance set at p-values<0.05. NS, no statistically significant differences detected; ^a^CVID group 1 vs HD; ^b^CVID group 2 vs HD; ^c^CVID group 3 vs HD; ^d^CVID group 4 vs HD; ^e^CVID group 1 vs CVID group 2; ^f^CVID group 1 vs CVID group 4; ^g^CVID group 2 vs CVID group 3; ^h^CVID group 2 vs CVID group 4; ^i^CVID group 3 vs CVID group 4.

As referred above, group 4 CVID patients presented clearly altered pre-GC B-cell maturation profiles, the most common alteration (31/31 cases) in their blood pre-GC B-cells consisting of absence/decreased number of cells at the earliest stages of maturation (stages 1-3). In addition, group 4 CVID patients showed abnormally lower counts in blood of CD5^-^ CD38^++^ CD21^het^ CD24^++^ -0.36 vs 0.98, 7.6, 1.6, and 0.89 cells/µl in group 1 (p=0.015), group 2 (p<0.0001) group 3 (p=0.003) patients and HD (p=0.004), respectively- and CD5^+^ CD38^+/++^ CD21^het^ CD24^++^ -1.4 vs 2.6, 12 and 5.6 cells/µl, group 1 (p=0.03), group 2, (p=0.0117) CVID patients and HD (p<0.0001)-, immature/transitional B cells, together with significantly higher counts of CD21^-^ CD24^-^ -9.9 vs 4, 0.74 cells/µl in CVID group 1 cases (p=0.0018) and HD, (p<0.0001) respectively- and CD21^-^ CD24^++^ mature naïve B-cells -3.5 vs 1.5 and 0.4 cells/µl, in group 1 patients (p=0.02) and HD, (p<0.0001) respectively-(**Table 1**). In addition, group 4 CVID patients also showed significantly increased blood counts vs HD of CD21^-^ CD24^-^ (1.15 vs 0.66 cells/µl, p=0.016) and CD21^-^ CD24^++^ (4.1 vs 1.18 cells/µl, p=0.003) MBC cells, compared to with the other CVID (groups 1–3) cases (**Table 1**).

From the clinical point of view, no major differences were observed among the patients included in the distinct CVID groups 1 to 4, except for a higher frequency of autoimmune cytopenias in group 4 patients vs. groups 1–3 cases (23% vs 6%, p=0.032), and a greater prevalence in group 2 patients of lymphadenopathy (63% vs. 15%, 33%, 23% in group 1, 3, and 4 cases, respectively; p=0.03) and systemic autoimmunity (25% vs. 0%, 16% and 3% in groups 1, 3, and 4, respectively; p=0.009) (**Supplementary Table 3**).

## Discussion

CVID is yet a poorly understood, clinically and prognostically heterogeneous disease, whose clinical management remains a challenge. Due to the lack of diagnostic genetic/molecular markers in the majority of patients, flowcytometric analysis of lymphocyte populations in blood, in combination with other laboratory data, has become critical for diagnosis and monitoring of CVID. Thus, at present it is well-established that simultaneously decreased plasmablast/plasma cell and/or switched-MBC counts in blood is a hallmark of CVID (8, 9, 12). Despite pre-GC B-cells appear to be preserved in a substantial proportion of CVID patients, an altered B-cell maturation in bone marrow together with reduced total B-cells, and an expansion of both immature B lymphocytes and CD21^low^ B cells in blood, have been recurrently reported in a subset of CVID patients (8, 13–16, 27, 28). Here, we investigated in detail the presence and frequency of alterations in both the numerical distribution and the immunophenotypic (maturation) profile of pre-GC B-lymphocytes based on a large cohort of 100 CVID patients from 8 different European centres in comparison to age-matched HD. For this purpose we used the standardized EuroFlow *pre-GC B-cell tube* and SOP (19–21) together with novel B-cell maturation software tools. Overall our results confirmed the presence of significantly reduced plasmablast/plasma cell counts in every CVID patient, most frequently associated with decreased switched-MBC. Despite total B-cell counts were significantly reduced in CVID vs age-matched HD, less than a third of all CVID patients investigated here showed abnormally decreased pre-GC B-cell counts in blood, in line with (extensive) previous observations by our and other groups (8, 9).

In order to gain further insight into the (potentially) altered pre-GC B-cell profiles in CVID, we dissected the pre-GC compartment into five distinct maturation-associated subsets of pre-GC immature and naïve B-cells. Overall, immature B cells were decreased in almost one third of the patients, while more mature naive B-lymphocytes were found to be below normal levels in only 11% of cases. Altogether these findings point out a defective B cell production and/or shortened survival of immature B cells (9) in a significant proportion of CVID cases. In line with this hypothesis, more in depth analysis of the immature and naive compartments of pre-GC B-cells showed that while <10% of CVID patients had lower counts in blood of the less differentiated CD5^-^ CD38^++^ CD21^het^ CD24 ^++^ immature B-lymphocytes, more than a third of the cases showed reduced counts of the more differentiated CD5^+^ CD38^het^ CD21^+^ CD24^+^ immature B-cells. In addition, this maturation blockade at the earliest stages of peripheral immature B-lymphocytes was associated with increased counts of the minor subsets of the more mature CD21^-^ (CD24^-^ and CD24^-++^) naive B-cells in virtually every CVID patient, as also extensively described previously by others in the literature (29–32). Thus, Vlková et al. (33) first reported that CD21^-^ B-lymphocytes expanded in CVID mostly comprise the CD21^-^ CD24^-^ and CD21^-^ CD24^++^ subsets. Despite the precise functional and clinical significance of both subsets still remains unclear, it has been previously shown that homeostatic stimulation of CD21^-^ B-cells induces proliferation of naive B-cells with loss of CD21 expression and differentiation toward CD21^-^CD24^-^ and CD21^-^CD24^++^ B-cells, in the absence of the classic GC reaction (33). However, in contrast to healthy individuals in whom the majority of CD21^low^ B-cells belong to the MBC compartment (29, 34), in CVID patients these cells have not undergone somatic hypermutation and show naïve B-cell features (35, 36) in line with our findings. Of note, expanded CD21^-^ B-cells have also been associated with T-bet^+^ B-cells in humans (37) and with interferon-gamma mediated dysregulation of B cell maturation induced by follicular helper T cells in CVID (38).

Normal differentiation and survival of immature/transitional and mature naive B-cells requires integration of distinct molecular signals after B-cells are released from the BM to the periphery (39). Thus, almost 40% immature peripheral blood B-cells produced in human bone marrow may be autorreactive (39, 40); at this stage, peripheral tolerance is checked and only a small percentage of all circulating immature B-lymphocytes will survive the negative selection and become mature naive B-cells (40). Immature B-cells retain low expression levels of BM-associated markers such as CD10, CD38, CD24 (41), and except for the less differentiated immature B-cells that are CD5 negative, they are also CD5^+^. In turn, recent BM emigrant immature B-cells express high levels of smIgM which contribute to the negative selection based on the B cell receptor (BCR) affinity for self-antigens (42). Further maturation of immature B-cells and mature naive B-cells is associated with loss of expression of CD38 and CD5, and down-regulation of CD24 levels (41, 43–45). CD38 has enzymatic activity and modulates peptide concentrations during B-cell signalling, and it is strongly expressed in the GC reaction where it prevents apoptosis of GC B-cells (43, 46). CD24 is also highly expressed on immature B-cells and it is associated with Siglec-10 that interacts with SHP-1 phosphatases, a complex implicated in NFκB activation and the prevention of signaling from non-specific antigen receptors (47). In turn, the CD21 complement receptor is part of the BCR co-receptor complex (CD19, CD21, CD81, and CD225) (48), that acts as positive regulator of antigen-mediated BCR signaling, a critical event for normal maturation of transitional B-cells (39) into naïve B-lymphocytes, and the differentiation of the later cells to MBC and plasma cells. Thus, CD21 reduces the B-cell activation threshold and contributes to amplification of BCR signaling (30). In contrast, CD5 is a negative regulator of BCR signaling that limits B-cell activation to avoid autorreactivity during the early steps of B-cell maturation in blood, while contributing to upregulate the BCR/TCR activation threshold after antigen recognition (44, 49, 50). Consequently, high levels of CD5 expression together with low CD21 expression on immature B-cells, avoid autorreactivity (43). Altogether, these findings support the notion that transitional/immature B-cells are particularly sensitive to subtle variations in stimuli and signals that determine cell death vs survival, on a delicate equilibrium that avoids excessive auto-reactivity while ensuring production of a sufficiently wide BCR repertoire, *via* further differentiation to mature naive B-lymphocytes. In addition, our findings about an altered numerical distribution of distinct maturation-associated subsets of immature B-cells and mature naive B-lymphocytes in CVID, suggest potential (co)existence of maturation blockades at the earliest stages of maturation of peripheral immature B-lymphocytes, with an abnormally increased differentiation toward less reactive CD21^-^ naive B-cells, that might finally lead to decreased plasma cell and switched memory B-cell counts in these patients. If this hypothesis holds true, an altered maturation of pre-GC B-cells would also be a hallmark of CVID, even among patients that show normal pre-GC B-cell counts.

In order to investigate in more depth the potential existence of an altered maturation of pre-GC B-cells in CVID toward CD21^-^ naïve B-lymphocytes, we built a normal (reference) database to trace the maturation of pre-GC B-lymphocytes from the less differentiated transitional/immature to the more mature naive B-cells. As expected, the normal (HD) database, showed that CD38, CD5, CD24, and smIgM are downregulated in the transition between immature/transitional B-cells and mature naive B-lymphocytes, in line with previous observations (42, 43). In contrast, comparison of individual CVID patients against the normal reference pre-GC B-cell maturation pathway, revealed altered marker expression profiles in every CVID patient analyzed, including four clearly different patterns of alteration. Thus, in around one third of the cases (CVID group 4) reduced numbers of immature/transitional B-cell subsets associated with increased counts of CD21^-^ naïve and unswitched MBC were found to be the consequence of a completely deregulated pre-GC B-cell maturation associated also with immunophenotypes for one or more markers that deviated significantly from those of normal pre-GC B-cells. Of note, among CVID group 4 patients, the altered immunophenotypic pre-GC B-cell maturation profiles and pre-GC B-cell counts were associated with a significantly higher frequency of systemic autoimmunity, as also reported by others (51), suggesting a potential failure of the negative selection of immature B-cells in BM, and a parallel attempt to keep B-cell production (and mature B-cell numbers) within the normal range. In contrast, pre-GC B-cell counts were less severely reduced in the other three CVID patient groups here identified, in line with the observation in these groups of patients of more conserved, normally-appearing, pre-GC B-cell maturation profiles. Despite this, multiple alterations on the expression levels of specific cell surface molecules along the pre-GC B-cell maturation pathway were observed in these patients, particularly in the CVID group 2 and group 3 here identified.

Similarly to CVID group 4 cases, group 1, and particularly group 2 CVID patients displayed overexpression of smIgM at intermediate stages of maturation of blood pre-GC B-cells, in association with significantly reduced switched MBC. In contrast, CVID group 3 cases showed both normal smIgM expression levels and closer to normal IgMD^-^ MBC counts. Downregulation of smIgM expression levels in B-cells at the latest stage of pre-GC B-cell maturation is required for further negative selection of autorreactive B-lymphocytes and adequate B-cell maturation. Thus, the overexpression of SIgM here observed might contribute to explain the higher frequency of autoimmune diseases in CVID group 2 patients, in line with previous findings by Warnatz et al. (27) in a subset (type Ia) of CVID patients, and also findings of other groups that showed high expression levels of smIgM and autoimmunity in PID other than CVID, reflecting B-cell activation (52) in these patients. This, together with the presence of higher levels of CD38 at intermediate stages of maturation of pre-GC B-cells, (which was also more pronounced in CVID group 2 patients), may reflect delayed maturation of immature B-cells to mature naive B-lymphocytes among this subgroup of CVID patients. This may contribute to explain why compared to the other CVID groups, CVID group 2 cases were also those who presented the highest counts of the more immature (CD5^-^ CD38^++^ CD21^het^ CD24 ^++^) blood B-cells. In parallel, lower levels of CD5 (found in common in the three CVID groups 1 to 3) together with a marked reduction of the amount of CD19 expressed/B-cell, particularly in CVID group 2 cases, may further affect the activation threshold (53) required for BCR-mediated B-cell activation and subsequent maturation of immature and naive B-lymphocytes to MBC and plasma cells in this group of CVID patients. In fact, reduced CD5 expression during pre-GC maturation might lower the BCR activation threshold responsible for the activation of autorreactive B-cells. This together with the increased numbers of CD21^-^ naïve B-cells would contribute to a lower B-cell (activation) response (36), leading to a more pronounced anergic state, as previously associated with CD21^-/low^ B cells in CVID (29, 30) and CD19-CD81 complex immunodeficiency (48), together with downregulation of B-cell activation receptors in parallel to upregulation of genes involved in the inhibition of B-cell proliferation (35). Interestingly, CVID group 2 cases showed partially overlapping clinical and immunological features to CVID Ia patients who are defined by coexistence of strongly decreased MBC and expanded CD21^low^ B-lymphocytes, and show a unique clinical behaviour with greater frequency (vs CVID Ib and II cases) of autoimmunity and organomegalies (8, 27). Despite these similarities, in our patients expanded CD21^-^ pre-GC B-cells were not exclusively detected among CVID group 2 patients, but they were also found in other patient groups, particularly in CVID group 4 cases. Further studies are required to better understand the relationship that might exist between both CVID patient groups.

In summary, detailed analysis of the maturation profile of pre-GC B-cells in CVID compared to normal blood B-cells, based on the Euroflow *Pre-GC B-cell* tube and maturation software tools, revealed systematically altered marker expression immunophenotypic profiles in CVID, in association with decreased pre-GC B-cell counts, with up to four distinctly altered profiles. Further studies in larger cohorts of CVID patients with longer follow-up are needed to confirm our results, and to correlate the distinct immune profiles here described with the underlying CVID-associated monogenic mutations and polygenic variant profiles, intravenous Ig therapy and patient outcome.

## Data Availability Statement

The original contributions presented in the study are included in the article/**Supplementary Material**. Further inquiries can be directed to the corresponding authors.

## Ethics Statement

The studies involving human participants were reviewed and approved by the local Ethics Committees of the participating centers: Hospital Universitario La Paz, Madrid, Spain (PI-2833 and 2009/3348/I); Charles University, Prague, Czech Republic (15-28541A); Erasmus MC, Rotterdam, The Netherlands (MEC-013-026); St Anne´s University, Brno, Czech Republic (METC 1G2015); BRC-Translational Immunology Lab, University of Oxford, Oxford, United Kingdom; University of Salamanca, Salamanca, Spain (USAL/CSIC 20-02-2013); University Hospital of Ghent, Belgium (B670201523515); and Faculdade de Medicina da Universidade de Lisboa and Centro Hospitalar Universitário Lisboa Norte, Lisbon, Portugal (937/13). The patients/participants provided their written informed consent to participate in this study.

## Author Contributions

EL-G, MB, TK, JP, AS, AO, and JD contributed to the conception and design of the study. LP-M, JTC, MP-A, EB, MW, CB, JN, TM, and A-KK performed data acquisition and patient data collection. LP-M, JTC, and MP-A performed data analysis. LP-M and QL built the pre-GC B-cell maturation database uploaded in Infinicyt. LP-M, EL-G, and AO wrote the manuscript. All authors contributed to the article and approved the submitted version.

## Funding

The coordination and innovation processes of this study were supported by the EuroFlow Consortium (Chairmen: MB and AO). LP-M was supported by FIS PI16/01605 and JTC by FIS PI13/02296 (Fondo de Investigación Sanitaria Instituto de Salud Carlos III, Madrid, Spain). The work was partially supported by grant PI20/01712-FEDER (Fondo de Investigación Sanitaria Instituto de Salud Carlos III, Madrid, Spain) and a grant from Fundación Mutua Madrileña (MMA, Madrid, Spain).

## Conflict of Interest

JD, MB, TK, MP-A, EL-G, A-KK, EB, and AO each report being one of the inventors on the EuroFlow-owned patent PCT/NL 2015/050762 (Diagnosis of primary immunodeficiencies), which is licensed to Cytognos, a company that pays royalties to the EuroFlow Consortium.

## Abbreviations

APS, automated populations separator; BCR, B-cell receptor; BM, bone marrow; CVID, common variable immunodeficiency; ESID, European Society for Immunodeficiencies; FCS, flow cytometry standard; FSC, forward light scatter; GC, germinal center; HD, healthy donor; Ig, immunoglobulin; IUIS, International Union of Immunological Societies; MBC, memory B-cell; MFI, mean fluorescence intensity; Pb, plasmablast/plasma cell; PC, principal component; PCA, principal component analysis; PID, primary immune deficiency; sm, surface membrane; SOP, standard operating procedure; SSC, sideward light scatter; WBC, white blood cell.
