# Supplementary material for: Dissection of the Pre-Germinal Center B-Cell Maturation Pathway in Common Variable Immunodeficiency Based on Standardized Flow Cytometric EuroFlow Tools
Source: Front Immunol. 2021 Feb 17;11:603972. doi: 10.3389/fimmu.2020.603972 (PMC7925888; doi:10.3389/fimmu.2020.603972)
Supplement: Supplementary file 1 [file DataSheet_1.pdf]

Supplementary tables and figures

Supplementary Table 1: Composition of the EuroFlow Pre-GC B-cell tube and information about the reference reagents included in it.

**Fluorochrome**	**BV421**	**BV510**	**FITC**	**PE**	**PerCP-Cy 5.5**	**Pe-Cy7**	**APC**	**APC-H7**
**Antibody**	CD27	IgM	CD38	CD5	IgD	CD19	CD21	CD24
**Clone**	M-T271	MHM-88	HB7	UCHT2	IA6-2	J3-119	B-ly4	ALB9
**Manufacturer**	BD Biosciences	Biolegend	BD Biosciences	Biolegend	Biolegend	Beckman Coulter	BD Biosciences	Beckman Coulter

Supplementary Table 2: Reference normal absolute B-cell subset counts as assessed in 62 healthy controls with a median age of 34y (range: 19-67y).

B-cell population	Nº of B cells/µl (range)
**Total B-cells**	206 (57-402)
**Immature/transitionals**	27 (5.7-126)
$CD5^- CD38^{++} CD21^{het} CD24^{++}$	0.89 (0.13-4)
$CD5^+ CD38^{+/++} CD21^{het} CD24^{++}$	5.60 (1.1-35)
$CD5^+ CD38^{het} CD21^+ CD24^+$	17 (4-94)
**Mature naive B-cells**	85 (24-203)
$CD21^+ CD24^+$	84 (23-201)
$CD21^- CD24^{++}$	0.74 (0.02-3.7)
$CD21^- CD24^-$	0.4 (0-0.14)
**Memory B-cells**	72 (19-159)
Memory $IgMD^+$	32 (7.6-88)
Memory $IgMD^-$	35 (11-90)

Results expressed as median cell counts per microliter of blood (range: 5th and 95th percentile values).

Supplementary Table 3: Frequency of distinct clinical manifestations of the disease in CVID patients grouped according to the pattern of alteration of pre-GC B-cell maturation.

**Clinical findings**	**CVID patient groups**				
	**Group 1 (n=42)**	**Group 2 (n=8)**	**Group 3 (n=7)**	**Group 4 (n=31)**	**p-value***
**Autoimmunity (AI)**					
Systemic AI	0%	**25%**	16%	3%	**0.009**
Organ based AI	17%	50%	17%	29%	NS
Cytopenias	7%	12%	0%	19%	NS
**Organomegalies**					
Lymphadenopathy	15%	**63%**	33%	23%	**0.03**
Splenomegaly	33%	71%	33%	43%	NS
Hepatomegaly	7%	28%	0%	6%	NS
**Tissue damage**					
LIP	2.5%	12%	0%	3%	NS
Granuloma	5%	0%	50%	7%	NS
Bronchiectasias	28%	62%	**80%**	45%	0.05
Enteropathy	17%	0%	57%	25%	NS

*Fisher exact test with significance set at p-values<0.05. NS, no statistically significant differences detected. AI, autoimmunity.

Supplementary Figure 1: Gating strategy used for the classification of circulating B-cell populations. In a first step, total B-cells were gated as $FSC^{lo}/SSC^{lo}/CD19^{+}$ (A). In a second step (B), B-cell were dissected into 9 subpopulations based on their staining profile for CD19, CD38, CD24, CD21, CD27, CD5, surface membrane IgM and IgD. Unsw: Unswitched. Sw: Switched.MBC: Memory B-cells. Pb: Plasmablasts. $IgMD^{+}$ MBC were further subdivided into $CD21^{+}$ $CD24^{+}$, $CD21^{-}$ $CD24^{++}$ and $CD21^{-}$ $CD24^{-}$ MBC.

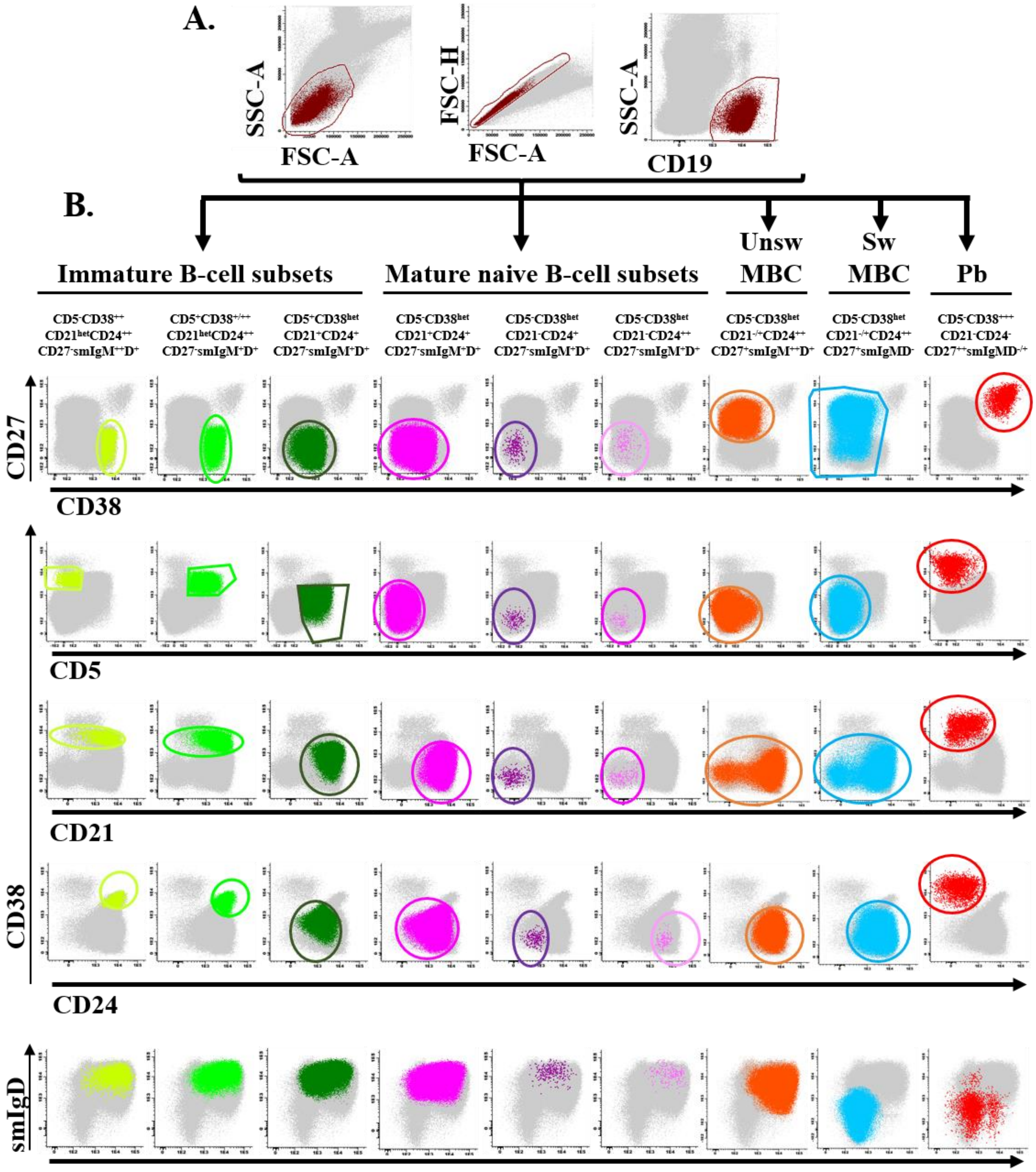

Supplementary Figure 2: Distribution of IgMD$^+$ MBC subsets in blood of CVID patients (n=100) vs age-matched HD (n=62). Results are shown using box and whiskers plots separately for HD (blue dots) and CVID patients (red dots) representing median and both the 5th and 95th percentile values. CVID patients showed reduced absolute numbers of CD21$^+$CD24$^+$ IgMD$^+$ memory B-cells, while they had normal absolute counts of CD21$^-$CD24$^-$ and CD21$^-$ CD24^{++} IgMD$^+$ memory B-cells (*** p-value<0.001, Mann Whitney U test).

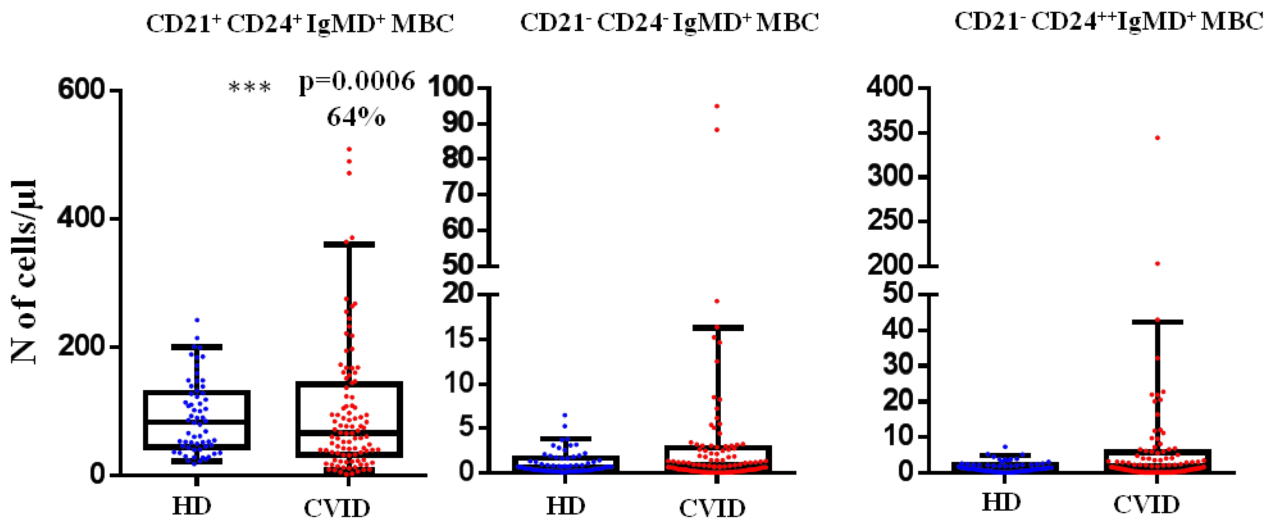

Supplementary Figure 3: Altered pre-GC B-cell maturation-associated phenotypes for individual markers representative of those three groups (clusters) of CVID patients (full lines) that showed overall normal-appearing pre-GC B-cell maturation patterns when compared to the reference HD cases (n=18); dotted lines represent 2SD values of normal reference mean fluorescence intensity values per marker and maturation stage.

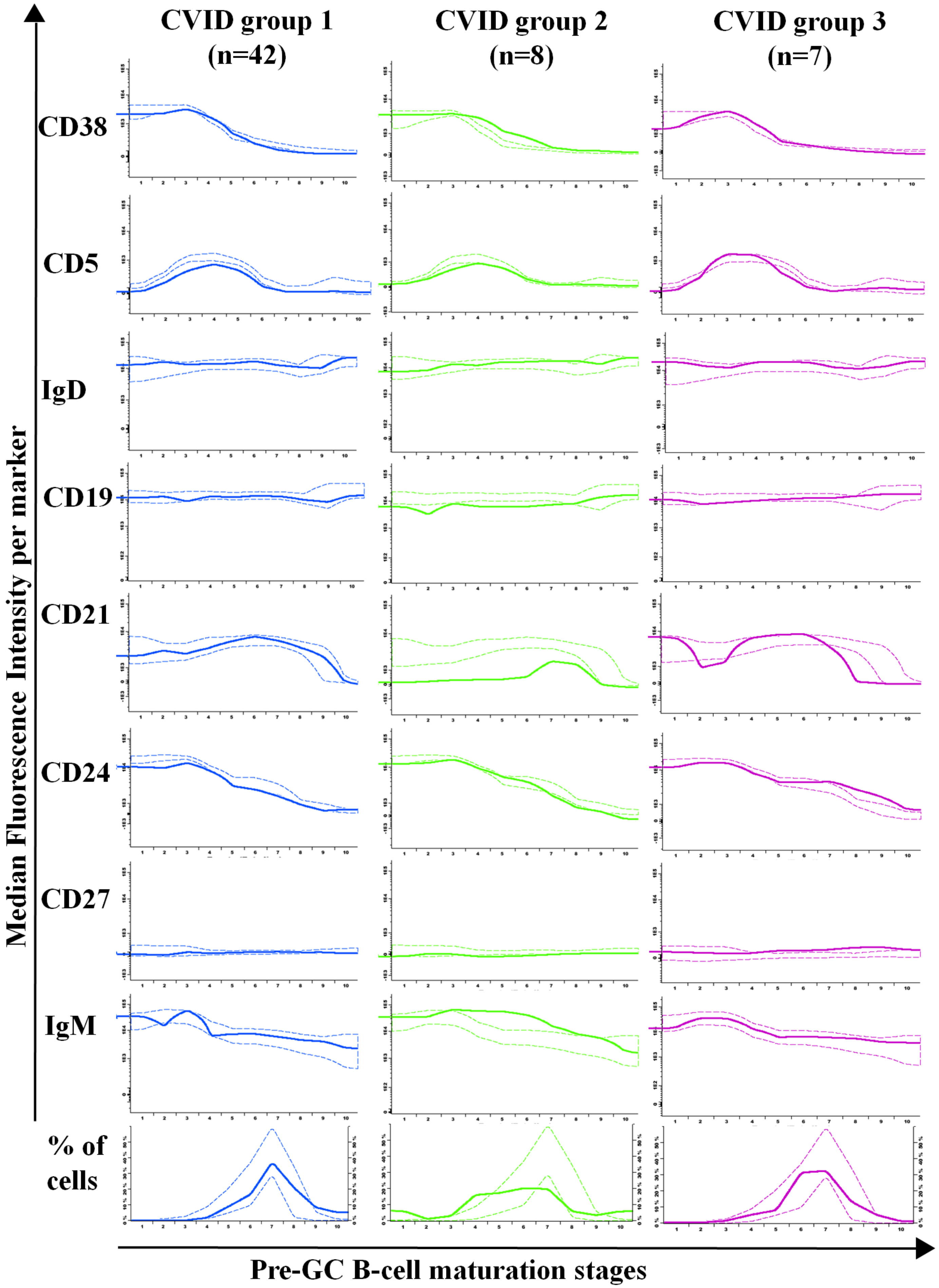

CVID group 1 (n=42)
CVID group 2 (n=8)
CVID group 3 (n=7)
CD38
CD5
IgD
CD19
CD21
CD24
CD27
IgM
% of cells
Median Fluorescence Intensity per marker
Pre-GC B-cell maturation stages
